# Primary care and mental health providers’ perceptions of implementation of pharmacogenetics testing for depression prescribing

**DOI:** 10.1186/s12888-020-02919-z

**Published:** 2020-10-28

**Authors:** Bonnie M. Vest, Laura O. Wray, Laura A. Brady, Michael E. Thase, Gregory P. Beehler, Sara R. Chapman, Leland E. Hull, David W. Oslin

**Affiliations:** 1 Department of Family Medicine, State University of New York- University at Buffalo Jacobs School of Medicine and Biomedical Sciences, Buffalo, NY USA; 2 VA Center for Integrated Healthcare, VA Western New York Healthcare System, Buffalo, NY USA; 3 VISN 4 Mental Illness, Research, Education and Clinical Center, Corporal Michael J. Crescenz VA Medical Center, Philadelphia, PA USA; 4 Department of Psychiatry, Perelman School of Medicine, University of Pennsylvania, Philadelphia, PA USA; 5 State University of New York- University at Buffalo, School of Public Health and Health Professions, Buffalo, NY USA; 6 VISN 4 Mental Illness, Research, Education and Clinical Center, VA Pittsburgh Healthcare System, Pittsburgh, PA USA; 7 Division of General Internal Medicine, Massachusetts General Hospital, Boston, MA USA; 8 Harvard Medical School, Boston, MA USA

**Keywords:** Implementation science, Pharmacogenetics, Depression, Veterans, Consolidated framework for implementation research

## Abstract

**Background:**

Pharmacogenetic testing (PGx) has the potential to improve the quality of psychiatric prescribing by considering patients’ genetic profile. However, there is limited scientific evidence supporting its efficacy or guiding its implementation. The Precision Medicine in Mental Health (PRIME) Care study is a pragmatic randomized controlled trial evaluating the effectiveness of a specific commercially-available pharmacogenetic (PGx) test to inform antidepressant prescribing at 22 sites across the U.S. Simultaneous implementation science methods using the Consolidated Framework for Implementation Research (CFIR) are integrated throughout the trial to identify contextual factors likely to be important in future implementation of PGx. The goal of this study was to understand providers’ perceptions of PGx for antidepressant prescribing and implications for future implementation.

**Methods:**

Qualitative focus groups (*n* = 10) were conducted at the beginning of the trial with Primary Care and Mental Health providers (*n* = 31) from six PRIME Care sites. Focus groups were audio-recorded and transcribed and data were analyzed using rapid analytic procedures organized by CFIR domains.

**Results:**

Analysis revealed themes in the CFIR Intervention Characteristics domain constructs of Evidence, Relative Advantage, Adaptability, Trialability, Complexity, and Design that are important for understanding providers’ perceptions of PGx testing. Results indicate: 1) providers had limited experience and knowledge of PGx testing and its evidence base, particularly for psychiatric medications; 2) providers were hopeful that PGx could increase their precision in depression prescribing and improve patient engagement, but were uncertain about how results would influence treatment; 3) providers were concerned about potential misinterpretation of PGx results and how to incorporate testing into their workflow; 4) primary care providers were less familiar and comfortable with application of PGx testing to antidepressant prescribing than psychiatric providers.

**Conclusions:**

Provider perceptions may serve as facilitators or barriers to implementation of PGx for psychiatric prescribing. Incorporating implementation science into the conduct of the RCT adds value by uncovering factors to be addressed in preparing for future implementation, should the practice prove effective.

**Trial registration:**

ClinicalTrials.gov ID: NCT03170362; Registered 31 May 2017

## Background

Pharmacogenetic testing (PGx) has potential to improve the quality of psychiatric prescribing by considering each patient’s unique genetic profile [1–3]. While routinely employed in some areas of medicine (e.g. cancer treatment), evidence that PGx can improve outcomes for patients with major depressive disorder (MDD) is limited [4]. MDD is challenging to treat and the likelihood of benefit decreases while the risk of treatment drop-out increases with each ineffective pharmacotherapy [5]. Using pharmacogenetics to guide medication selection has the potential to enhance patient engagement, reduce side effects, and improve disease outcomes and patients’ quality of life [6, 7].

Although psychiatric PGx tests are commercially available and covered by some insurance plans, there is limited evidence demonstrating their effectiveness for improving MDD [4, 8]. Previous studies have enrolled small samples of patients and produced mixed results, with some demonstrating improvement in depression while others found reduced side effects, but no change in efficacy [1, 9–11]. A recent meta-analysis concluded that pharmacogenetic-guided treatment for depression improved response and remission rates, but noted many limitations [4].

Given insufficient evidence to support widespread use, but a potentially large impact on patient care, the Veterans Health Administration (VHA) Precision Medicine in Mental Health Care (PRIME Care) study was designed to clarify the clinical utility of a specific commercially-available pharmacogenetic (PGx) test to assist treatment of MDD compared to usual care, and to identify how healthcare systems may best implement this novel practice. Work examining provider perceptions of PGx testing for psychiatric conditions has been limited [12–15]. Providers felt testing lessened patient resistance to medications by potentially reducing side effects and reassuring patients, but were concerned about managing patients’ expectations [15]. Baseline surveys conducted with providers engaged in PRIME Care indicated that > 85% of providers had never ordered a genetic test to assist with psychiatric prescribing, and providers reported limited training in genetics or access to genetic expertise [16].

Implementation science examines the broad range of contextual factors that affect the use of evidence-based practices in every day settings [17]. Incorporating qualitative inquiry within clinical trials helps to “capture greater diversity and depth of data on program outcomes,” and to better understand the experiences of trial participants [18]. Given the pragmatic nature of PRIME Care, implementation science methods provide a valuable opportunity to understand contextual factors which may be important for future implementation.

Implementation science activities were based on the Consolidated Framework for Implementation Research (CFIR) [17, 19]. This framework organizes factors important to implementation into five domains (Intervention Characteristics, Individual Characteristics, Inner Setting, Outer Setting, and Implementation Process) [17]. Implementation science activities are conducted in two phases. The first phase, reported here, captured providers’ perceptions of PGx for antidepressant prescribing prior to their experience with the test, and is focused on their perceptions in the domain of Intervention (PGx) Characteristics. We describe, using qualitative methods, providers’ perceptions around the use of PGx testing for MDD prescribing at the beginning of PRIME Care, to understand how these perceptions may relate to potential barriers and facilitators to future implementation. If the trial data support broader use of psychiatric PGx testing, these lessons will be critical for broader implementation in VHA. The second phase, to be conducted late in the trial, will focus on the other CFIR domains (Individual Characteristics, Inner Setting, Outer Setting, and Implementation Process) and will examine providers’ experiences using the PGx testing during the study, barriers and facilitators they encountered, and their recommendations for future implementation.

## Methods

Qualitative focus groups were conducted early in the trial. All study procedures were approved by the VHA Central IRB, as well as the appropriate oversight bodies at each of the participating sites. All participants provided written consent to participate in the PRIME Care study, including the focus groups.

### Study context and setting

PRIME Care is a 22 site, pragmatic RCT, conducted within VHA primary care (PC) and mental health (MH) outpatient clinics. PC and MH providers were recruited to participate in the trial. Providers then referred eligible patients to the study team; the first patient was randomized in August 2017. Randomization occurred at the patient/provider dyad level; each dyad received PGx testing results either immediately (~ 2–5 days after enrollment) or 6 months later (delayed group). Study outcomes include whether the immediate PGx test result dyads have significantly better treatment outcomes (i.e., improved PHQ-9 scores, remission of depression) and to what extent prescribers used PGx information when selecting an antidepressant.

### Recruitment

Study sites began enrolling provider participants in July 2017. Eligible prescribing providers (MD, DO, PA, or CRNP) were outpatient providers in mental health clinics and primary care clinics. Providers were classified as PC or MH based on their specialty training, their practice location, and scope of practice within the VA. During provider enrollment each site was responsible for introducing the study and the specific PGx test being used. The speed of provider enrollment varied across sites; therefore focus groups were conducted at sites where a sufficient number of both MH and PC providers were enrolled at the time of data collection. Within these sites, local site investigators recruited a convenience sample from enrolled and consented providers to participate in site-based groups.

### Data collection

At enrollment, providers completed a demographic questionnaire which assessed their specialty, practice location, years in practice, age, race/ethnicity, and gender.

Focus groups were conducted virtually through the VHA tele-conferencing system allowing the same team of interviewers to facilitate all groups. Focus groups were conducted between December 2017 and May 2018 before most providers had any experience with referring patients and using the test results. Participants could call in separately using their individual workstations. At four sites, two separate focus groups were held, one for MH and one for PC providers. The fifth site hosted one focus group for MH providers, and then due to insufficient numbers of available PC providers at that site, a sixth site was added and held one group for PC providers. Participants were verbally reminded of consent information at the beginning of the focus group session.

The sessions were facilitated by the implementation science leads (LW, SC), with a research assistant to manage logistics. Each session began with a brief presentation on pharmacogenetic testing, including a sample report, discussion of how results might be interpreted, and a brief question and answer period. This presentation was provided by PRIME Care leadership (DO, MT), experts in pharmacogenetics evidence and practice. These experts then left the call and audio recording began. Focus groups were conducted by a PhD-level medical anthropologist (BV) trained in qualitative health services research. The interviewer had no prior relationship with participants.

Focus group questions (Table 1) were developed based on CFIR Intervention Characteristics constructs, including: Intervention Source, Evidence, Relative Advantage, Adaptability, Trialability, Complexity, Intervention Design, and Cost. Feedback from key study stakeholders (study leadership, advisory board, and local site investigators) identified priority constructs within the Intervention Characteristics Domain. Stakeholders ranked each construct’s importance to PGx implementation on a Likert Scale and participated in an open-ended discussion during an in-person study kick-off meeting. Stakeholder feedback emphasized the constructs of Evidence, Relative Advantage, and Complexity as the most important areas to cover during the discussion and focus group questions were targeted accordingly, but contained flexibility to allow other constructs and topics to arise.

**Table 1:** Focus group questions

Question	CFIR Construct
1. Prior to deciding to enroll in this study, describe your knowledge of, and experience with, pharmacogenetic (PGx) testing.	Evidence
2. Based on your understanding of the literature, tell me your thoughts on the strength of the evidence behind PGx testing? a. What are your thoughts on how PGx testing might help you or your patients?	Evidence
3. How do you feel that PGx testing compares to your usual approach to prescribing for depression? a. Do you see any advantages? Why or why not?	Relative Advantage
4. To what extent do you feel you will incorporate the feedback from PGx testing in your prescribing for depression? b. What factors do you foresee influencing this?	Relative Advantage
5. Specific to the PGx reports used in this study, what do you *like* about these reports? What do you *not like?* a. Are there other report formats you have seen, aside from the one we are using in this study? If so, what are your thoughts on how they compare?	Complexity, Intervention Design
6. What barriers do you foresee to using PGx for depression prescribing? Do you have any concerns about using PGx*?*	Complexity
7. What would help you to best use the PGx testing results?	Multiple
8. Is there anything else related to the PGx testing/ study intervention that we haven’t discussed already that you feel is important?	Multiple

### Data analysis

Focus group audio recordings were transcribed verbatim by the VA Centralized Transcription Services Program. Transcripts were analyzed using rapid analytic procedures [20, 21]. This approach was chosen so that the resulting findings could inform study implementation by identifying early factors (e.g., provider attitudes, perceptions, knowledge about the intervention) that may affect provider recruitment, retention, and engagement with the trial [20]. Rapid analysis has been shown to generate results and interpretations comparable to traditional in-depth qualitative analysis methods [21]. Two primary analysts (BV, LB) reviewed each transcript and independently completed a template in Microsoft Excel, summarizing the findings in each area. The analysts then compared summaries and developed a final comprehensive summary for each group. Each summary contained an overall synopsis, an identification of key themes, and exemplary text and quotations from the transcripts. Each theme was then coded by CFIR construct. Each summary was reviewed with its transcript by an additional analyst (SC, GB, LH) to ensure completeness and accuracy. Any questions were resolved by the two primary analysts through discussion and refinement of definitions.

Finally, the group summaries were combined into an overall spreadsheet, organized by the CFIR Intervention Characteristics constructs, with each groups’ findings in a separate column. This matrix facilitated comparison of findings across groups and MH and PC providers. All authors met to review and discuss the analysis and final interpretation of the results.

## Results

### Participants

Ten focus groups (5 primary care, 5 mental health) were conducted with providers (*n* = 31) from 6 sites. The number of participants per group ranged from 1 to 5 with an average of 3.1. One scheduled group had only one attendee, therefore an individual interview was conducted. Focus groups lasted approximately 45 min.

As intended, providers were approximately half MH (48.4%) and half PC (51.6%). 61.3% of participants completed their medical training after 2000. Internists and psychiatrists represented 38.7% of participants each, 3.2% were family medicine, and 16.1% were Nurse Practitioners or Physician Assistants. Additional participant characteristics are presented in Table 2. During the focus groups, most providers stated they had not yet referred any patients to the study.

**Table 2:** Provider demographics

Characteristic	% (N)
Provider type	
Primary care	51.6 (16)
Mental health	48.4 (15)
Year training completed	
Before 1980	3.2 (1)
1981–1990	22.6 (7)
1991–2000	9.7 (3)
After 2000	61.3 (19)
Profession	
Internist	38.7 (12)
Family medicine	3.2 (1)
Psychiatrist	38.7 (12)
Physician assistant	3.2 (1)
Advanced practice nurse	12.9 (4)
Location of practice within VA	
Primary care @ medical center	48.4 (15)
PC mental health integration @ medical center	6.5 (2)
Mental health @ medical center	32.3 (10)
Mental health @ community clinic	9.7 (3)
Percent of time in clinical practice	
< 10%	3.2 (1)
25–49%	16.1 (5)
> 50%	77.4 (24)
Sex	
Male	48.4 (15)
Female	48.4 (15)
Missing	3.2 (1)
Age	
31–40	29.0 (9)
41–50	25.8 (8)
51–60	29.0 (1)
60+	12.9 (4)
Missing	3.2 (1)
Race	
White	74.2 (23)
African-American	6.5 (2)
Asian	12.9 (4)
Hispanic	3.2 (1)
Other	3.2 (1)

### Qualitative findings

Focus group analysis revealed themes within the CFIR Intervention Characteristics domain constructs of Evidence, Relative Advantage, Adaptability, Trialability, Complexity, and Intervention Design that are important for understanding providers’ attitudes and perceptions around implementation of PGx testing for depression prescribing (Table 3).

**Table 3:** Primary sub-themes within each CFIR construct

CFIR construct(s)	Main sub-themes
Evidence	• Limited knowledge of evidence • Evidence not yet definitive • Cautious about using until more evidence
Relative advantage	• Hopeful, but unsure • May be especially useful for patients with prior unsuccessful treatment • Just one additional piece of information • Concern over delay compared to usual practice
Adaptability, Trialability, Complexity	• Concern over workflow and time to discuss/ educate the patient • Primary Care providers concerned over how would fit with usual scope of practice • Desire to try it out with some patients, or tailor use based on patient characteristics
Intervention Design	• Simple to use • Concern over misinterpretation of colors/ categories

#### Evidence

Evidence is used to describe “stakeholders’ perceptions of the quality and validity of evidence supporting the belief that the intervention will have desired outcomes.” [22] Providers discussed limited knowledge of the evidence around PGx testing for depression. As one MH provider stated,

“*I think it definitely would be a great guide. And I’m still not as convinced with the strength of the evidence, but I’d like to see it a lot more.*” [MH Group]

While generally not familiar with the literature, providers did have a general understanding that the evidence for psychiatric prescribing was not yet definitive and were therefore uncertain of the clinical utility of the PGx testing. One MH provider expressed concern, but was hopeful,

*“I am concerned because a lot of genetic studies have been really, really promising in the past, but haven’t really panned out in terms of helping clinically to a great extent. So, kind of cautiously optimistic, I’ll take that data that we get at the end of this study and see really how it matches up.”* [MH group]

PC providers were comfortable with and knowledgeable about the level of evidence for genetic testing in other areas of medicine (e.g., for anti-platelet medications and genetic susceptibility to disease). They expressed concern that the evidence for PGx in depression prescribing was limited and were therefore hesitant about implementing the intervention.

*“I mean warfarin is the one that’s the most well defined, but really it hasn’t changed anybody’s practice particularly. And there’s also ones for [drug name], but as far as I know, nobody actually uses that in their decision making at least here at the VA, so, I guess I’d say we need more evidence as to whether this is clinically useful or not.”* [PC Group]

As a result of this general uncertainty around the evidence, providers expressed caution about PGx testing. They discussed not giving the test results much weight until there is more evidence and they have clinical experience with the test.

*“I don’t know that I know enough about the strength. I think there’s still a lot of unanswered questions and for me, understanding what is the incremental value of having this is still unclear … there’s not enough evidence to support its widespread use … I’ll wait until the evidence tips more in favor and is a little more concrete.”* [PC Group]

#### Relative Advantage

Relative Advantage refers to “stakeholders’ perception of the advantage of implementing the intervention versus an alternative solution” or usual practice [22]. Given limited evidence, participants were also unsure whether PGx testing would improve upon their current practice. However, providers expressed interest in the potential, and were hopeful that PGx testing would help them identify more effective medications for their patients.

*“I think the advantage is hopefully avoiding a little bit of that trial and error that we always have.”* [PC Group]

Providers saw PGx testing as providing an advantage over usual care for patients who have been difficult to treat or have had poor responses to medication.

*“Especially I think patients with multiple comorbid medical conditions … just getting a sense some patients who are fast metabolizers and they do need higher doses of the medications and stuff like that. I think that will kind of open doors to having a better understanding of their medication regimen.”* [MH Group]

Many commented that this approach could increase patient buy-in to the use of medication to treat their depression and help overcome patient resistance or concerns around medication and possible side effects.

*“If a patient believes that this [has] been this process to help them choose the right medication and initially creates some positive placebo effect until the medication’s real effect kicks in … if you can create a positive experience with the medication they’re more likely to be med compliant.”* [MH Group]

Generally, participants saw PGx testing as one additional piece of information to add to other factors that impact their decisions about depression medication, but were unsure how much relative weight it would have. As one provider expressed,

*‘The fact that psychiatry is touched by so many different components of a human person … So we’re looking at the genes as if they are the sixth unmovable set of dictates that kind of tells you what is going to happen to a person. And yet then, we have the nun study that they’ve got 97-year-old nuns with the APOE gene and have never demonstrated any symptoms of Alzheimer’s … So hanging all of our hats on these hooks may not be where it’s at in terms of fixing the problem of psychiatry not making people better. But I’m hoping that it can give us one more tool to use wisely and judiciously.”* [MH Group]

Participants also expressed concerns over the inherent delay in prescribing due to the wait for PGx test results compared to usual practice. Rather than writing a prescription at the appointment, ordering the test requires waiting up to 3 days and re-contacting the patient, which providers felt might lead to a missed treatment opportunity.

*“The time waste, sort of the point of care act timeliness is probably the biggest area I would say … assuming that I fully buy-in and I fully believe the evidence, and there’s clear support for this being of incremental value, I think the process barrier that I see to actually using it is the timeliness because if you’ve got a patient who is there with you and going to pick up the meds, you don’t want to lose that opportunity.”* [PC Group]

#### Adaptability, Trialability, and Complexity

Focus group conversations related to the CFIR constructs of Adaptability, Trialability, and Complexity contained significant overlap, and therefore are discussed together. Within CFIR, Adaptability refers to “the degree to which an intervention can be adapted, tailored, refined, or reinvented to meet local needs,” while trialability is defined as “the ability to test the intervention on a small scale in the organization, and to be able to reverse course (undo implementation) if warranted.” [22] Complexity is, “the perceived difficulty of the intervention, reflected by duration, scope, radicalness, disruptiveness, centrality, and intricacy and number of steps required to implement.” [22]

As mentioned, participants expressed concern over how PGx testing will fit in to the workflow for depression treatment, seeing it as potentially difficult to adapt to existing practices. In addition to the potential prescribing delay, providers were concerned about complex discussions of PGx, especially with patients with new depression or patients who were resistant to treatment. This concern was prominent among the PC providers, who noted that depression is not usually the primary or only complaint they address within a patient visit. As one provider described:

*“It’s the uncommon patient who comes in and says my chief complaint is that ‘I’m depressed and I want you to help me with it.’ It’s usually something that trickles out two-thirds of the way through a visit for routine care. And it’s a long conversation to begin with in terms of assessing how they’re doing, how severe their problem is, are they suicidal, where is their interest in seeking treatment or other kinds of modalities for care … I’m wondering where I’m going to squeeze the rest of this in.”* [PC Group]

Participants in other groups echoed similar concerns around fitting time in to discuss PGx testing and provide education to the patient, indicating that they would not use the testing depending on those circumstances.

*“Where this makes the most sense to me is in patients that have familiarity with medications for depression, prior trials that either worked well for a time and then became less effective or ineffective or had problematic side effects in the outset. I think it is more challenging in the context of a discussion about 'What is depression? What are medications for depression?' with somebody who is new to both questions.”* [PC Group]

PC providers also expressed concern over their familiarity with many of the medications in the report, and their ability to adapt the test results. One provider mentioned,

*“There’s a comfort level we have, we have a usual repertoire and beyond that there’s also, some of it maybe that we don’t use as often, but we do have a comfort level with. And there were some that we would never use. If I remember correctly, I think that the ones that were maybe most recommended were things that I would never consider using. And I use one, something that was sort of in the middle row that seemed like it would be helpful. So I think it does help, at least gives us food for thought … there still are some things that we would probably never feel that comfortable prescribing.”* [PC Group]

Due to these concerns, providers discussed a preference for implementing PGx slowly, testing out the process in a few patients (i.e., trialability). They also discussed how the decision to introduce PGx testing would depend on other factors they are addressing with a patient (i.e., adaptability).

*“How we decide to prescribe the medication always depends on what are the exact factors we’re targeting. Either we want the patient to sleep more, or sleep less. You know their safety profile for that patient, interaction with other medication, so I think this will be an important tool to kind of add to those factors when prescribing the medication.”* [MH Group]

#### Intervention Design

The CFIR construct of Intervention Design refers to “perceived excellence in how the intervention is bundled, presented, and assembled.” [22] During the focus groups, we asked providers to reflect on the layout, format, and use of the PGx test result reports, which provide a list of medications categorized into columns by green (no drug-gene interactions), yellow (possible drug-gene interaction) and red (serious drug-gene interaction), with footnotes that provide guidance on potential prescribing modifications. Many of the comments around design also reflected the complexity of the PGx testing.

Providers generally felt the report was simple to use and that the color-coding of medications was straightforward. As one provider said,

*“I think the simple categorization is useful just because I myself do not want to spend a lot of time thinking about this, right? And so having a relatively simple … the sort of green light, yellow light, red light is fine. I mean in one way what it's analogous to is the CDC immunization schedule … I think having sort of a two-level thing where I can get a quick answer and if I need more I can look and get more, to me is a good way of doing it*.” [PC Group]

However, both MH and PC providers expressed concern over the potential for misinterpretation related to the color coding, especially by patients.

*“I guess if there was some reason that there was a particular medication that we really wanted to use and it was on the yellow or the red list and then the patient is saying well, ‘why would you want to put me on something that’s on the yellow or the red?’ just kind of looking at it. And even if in my clinical judgment I thought for other reasons that they were the most appropriate medication, I could see that that could kind of complicate the conversation.”* [MH Group]

*“So I think that liability is a concern … you know if there is an adverse reaction … I’m concerned that if there’s some formal piece of information that says, hey this guy had this adverse outcome and he was on a medicine that was … “use with extreme caution”, I’m a little nervous about that but maybe that’s an unrealistic concern to have. But unlike clinical judgment where it’s easy to sort of you know, defend that, this seems kind of black and white, or red … I’d be less concerned about us misusing it than the misperception from other people.”* [PC Group]

## Discussion

Focus group results demonstrated the applicability of the CFIR constructs for understanding factors that may affect use of PGx testing and the context for broader dissemination. Providers expressed concerns about the intervention itself in terms of the evidence supporting it, the relative advantage compared to usual practice, and the feasibility of using PGx testing in daily practice. These concerns about the strength of the evidence are expected, as PRIME Care is a hybrid type 1 trial [23], developing the evidence for PGx testing while simultaneously studying its implementation. Providers’ hesitation around PGx testing points to the need during the trial to remind providers that their participation is an important step towards contributing to the evidence and determining whether widespread use is justified. Further, understanding providers’ baseline knowledge and attitudes about PGx testing will provide insight into barriers to be addressed in future clinical implementation should the trial demonstrate the clinical utility of PGx.

While our questions were targeted to elicit responses pertinent to constructs within a specific CFIR domain (Intervention Characteristics), participants’ view of their context resulted in broader discussions. For example, the needs and opinions of those served by the organization, i.e., the patients (CFIR domain of Outer Setting), were frequently raised as a factor in providers’ thoughts about how they may use PGx testing. Providers felt some patients would be more appropriate than others based on their characteristics and needs.

Our findings also revealed aspects which cut across CFIR constructs that are important to understand in preparing for future implementation. First, providers had limited experience and knowledge of PGx testing and its evidence base, particularly for psychiatric medications. While providers held favorable attitudes towards PGx testing, they wanted additional information on the evidence and the clinical utility. Second, providers were hopeful that PGx could increase their precision in prescribing antidepressants and improve patient engagement, but were uncertain how much results would influence treatment. Participants were mixed as to how helpful they felt this extra information would be. Third, providers indicated some serious concerns about potential misinterpretation of PGx results, especially on the part of patients who might not understand the report’s nuances. They also had significant concerns about incorporating testing into their workflow, given the delay in receiving the results and potential difficulties around re-engaging patients. These findings are consistent with quantitative data on the full sample of PRIME Care participating providers [16] as well as those concerning the use of PGx more broadly [12–15]. Most studies have used quantitative surveys to examine these attitudes; the use of focus groups and qualitative data allow us to understand and explore the context underlying providers’ thoughts on PGx, which help identify modifiable factors to be addressed in implementation.

The PRIME Care trial offers a unique opportunity to examine differences between MH and PC providers’ use of PGx testing in the context of depression care. Previous studies have documented familiarity, comfort, and use of PGx broadly among primary care providers, but have not focused specifically on psychiatric prescribing [12, 15]. Our results demonstrate that PC providers were less familiar and comfortable with the application of PGx testing to antidepressant prescribing than MH providers, and may be less comfortable with the range of medications available in the report. This finding suggests that additional support may be needed in PC settings. Within the VHA, such assistance is available via integrated mental health primary care models, but may be more difficult to access in systems where integration is not available. Given variable comfort and confidence in dealing with depression in primary care settings [24] and variations in the use and perceived value of integrated mental health care [25], it is important to understand how PC providers can be supported to use novel resources and tools such as PGx testing to improve PC management of depression. In the event that the trial produces a negative result and does not show PGx to be effective, the insights gained into how PC and MH providers approached the use of PGx testing for antidepressant prescribing will likely be applicable to the implementation of other new psychiatric practices.

### Limitations

Limitations of this study include generalizability. As a qualitative study, these results may not be generalizable to all providers. The sample was drawn entirely from the VHA, which may have resulted in different contextual factors being emphasized. For example, cost of the PGx testing was not a focus in these discussions, but may be important in the private sector. Further, the sample of providers may be subject to volunteer bias, as they already consented to participate in a PGx trial and may have different views from those who chose not to participate. However, the enrolled providers may also represent PGx enthusiasts who would be most willing to use PGx testing in their clinical practice; therefore, the volunteer bias may mirror real-life individual predispositions towards early adoption. The PRIME Care study examines the use of one specific commercially-available PGx test report, and results may not be generalizable to all currently available PGx testing. Finally, focus group questions targeted a particular CFIR domain (Intervention Characteristics). Other CFIR domains may be equally salient, and will be studied in ongoing implementation science activities during other phases of the study.

## Conclusions

Overall, our findings demonstrate possible barriers and facilitators to be considered in future implementation of PGx testing for depression. Phase 2 of the PRIME Care implementation science activities will ask providers to reflect on other CFIR domains, including Individual Characteristics, Inner Setting and Outer Setting, and Implementation Process in order to understand their experience in the trial, explore their use of the PGx testing, barriers and facilitators encountered in the process, and solicit thoughts on clinical implementation. These future data will provide insight into how perceptions and attitudes around PGx testing at the beginning of the trial relate to actual use of the test, and implications for future clinical implementation of this novel psychiatric practice.

## Data Availability

De-identified data from the transcripts available from the corresponding author on reasonable request.

## Abbreviations

CFIR: Consolidated Framework for Implementation Research
MDD: Major depressive disorder
MH: Mental Health
PC: Primary Care
PGx: Pharmacogenetic testing
RCT: Randomized controlled trial
VHA: Veterans Health Administration
